# Supplementary material for: Palygorskite-Based Organic–Inorganic Hybrid Nanocomposite for Enhanced Antibacterial Activities
Source: Nanomaterials (Basel). 2021 Nov 28;11(12):3230. doi: 10.3390/nano11123230 (PMC8707592; doi:10.3390/nano11123230)
Supplement: Supplementary file 1 [file nanomaterials-11-03230-s001.zip › nanomaterials-1465549-supplementary.pdf]

nanomaterials

Supplementary Materials

Palygorskite-Based Organic–Inorganic Hybrid Nanocomposite for Enhanced Antibacterial Activities

Aiping Hui, Fangfang Yang, Rui Yan, Yuru Kang and Aiqin Wang *

Key Laboratory of Clay Mineral Applied Research of Gansu Province, Center of Eco-Material and Green Chemistry, Lanzhou Institute of Chemical Physics, Chinese Academy of Sciences, Lanzhou 730000, China; aphui1215@163.com (A.H.); yangff@licp.cas.cn (F.Y.); yanjie4712010@163.com (R.Y.); yurukang@licp.cas.cn (Y.K.)

* Correspondence: aqwang@licp.cas.cn; Tel.: +86-931-4968-118

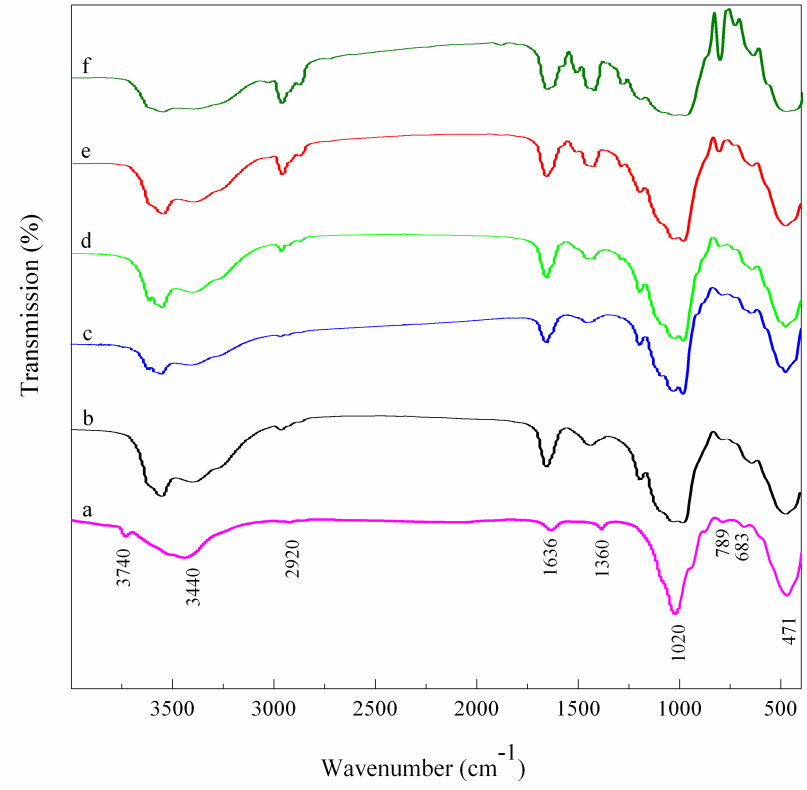

Figure S1. FTIR spectra of PAL encompassed by (a) 0%, (b) 1%, (c) 2.5%, (d) 5%, (e)10%, (f) 20% CAR.

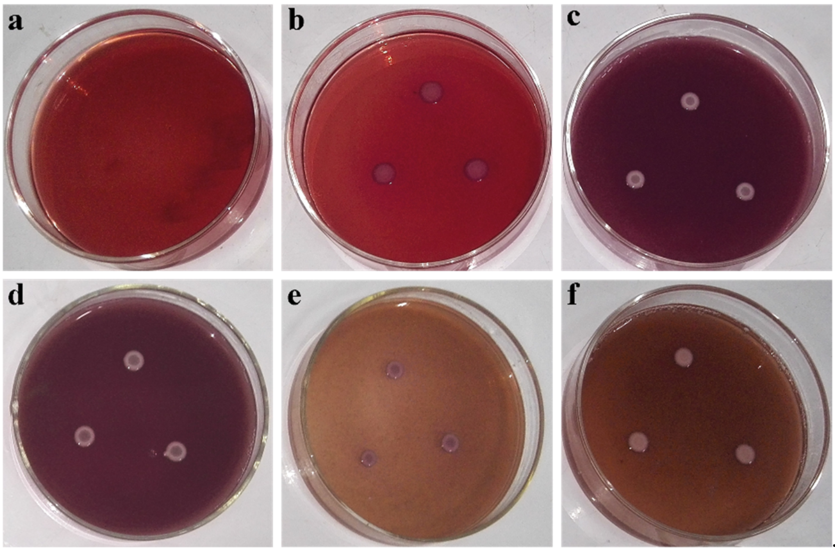

Figure S2. (a) Blank control, (b) positive control of *E. coli*, *E. coli* treated by PAL with different concentrations (c) 50 mg/mL, (d) 20 mg/mL, (e) 10 mg/mL, (f) 1 mg/mL.

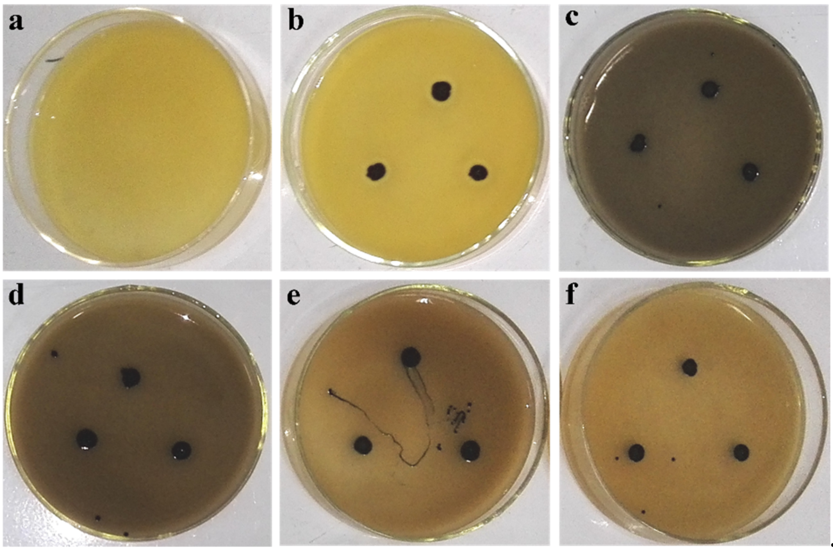

Figure S3. (a) Blank control, (b) positive control of *S. aureus*, *S. aureus* treated by PAL with different concentrations (c) 50 mg/mL, (d) 20 mg/mL, (e) 10 mg/mL, (f) 1 mg/mL.

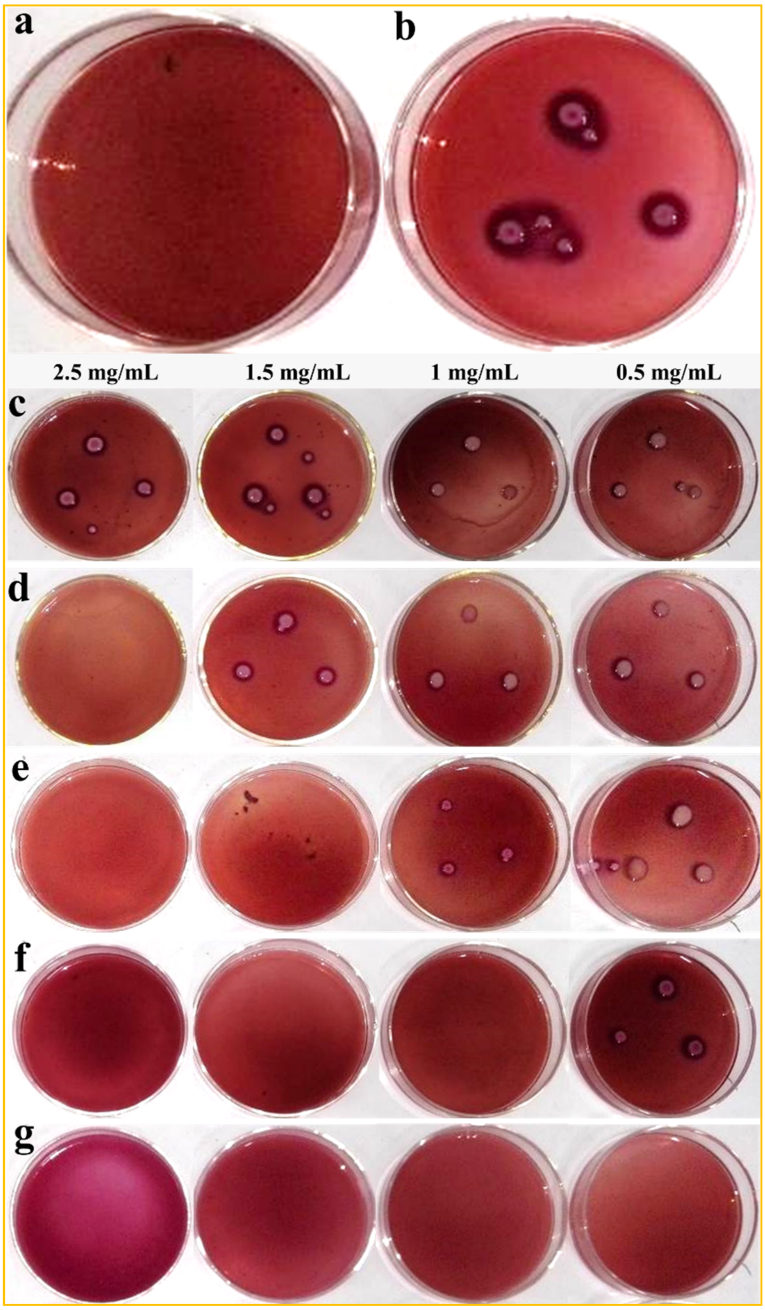

Figure S4. (a) Blank control, (b) positive control, MIC evaluation of PAL encompassed by (c) 1%, (d) 2.5%, (e) 5%, (f)10%, (g) 20% CAR.

Table S1. Effects of preparation parameters on antibacterial activities of ZnO/PAL against *E. coli*.

Factors	Alkali	$n_{Zn^{2+}}/n_{alkali}$	Temperature (°C)	Time (h)	PAL (%)	MIC (mg/mL)
S1	$NH_3 \cdot H_2O$	1:3	450	4	20	5
S2	NaOH	1:3	450	4	20	5
S3	HMTA	1:3	450	4	20	10
S4	Na_2CO_3	1:3	450	4	20	10
S5	NaOH	1:1	450	4	20	10
S6	NaOH	1:1.5	450	4	20	10
S7	NaOH	1:2	450	4	20	5
S8	NaOH	1:2.5	450	4	20	5
S9	NaOH	1:3	450	4	20	10
S10	NaOH	1:2	300	4	20	5
S11	NaOH	1:2	350	4	20	2.5
S12	NaOH	1:2	400	4	20	2.5
S13	NaOH	1:2	450	4	20	5
S14	NaOH	1:2	500	4	20	5
S15	NaOH	1:2	600	4	20	10
S16	NaOH	1:2	400	1	20	5
S17	NaOH	1:2	400	2	20	5
S18	NaOH	1:2	400	3	20	2.5
**Factors**	**Alkali**	$n_{Zn^{2+}}/n_{alkali}$	**Temperature (°C)**	**Time (h)**	**PAL (%)**	**MIC (mg/mL)**
S19	NaOH	1:2	400	4	20	2.5
S20	NaOH	1:2	400	6	20	5
S21	NaOH	1:2	400	8	20	5
S22	NaOH	1:2	400	3	10	1.5
S23	NaOH	1:2	400	3	20	1.5
S24	NaOH	1:2	400	3	30	2.5
S25	NaOH	1:2	400	3	40	5
S26	NaOH	1:2	400	3	50	10
S27	NaOH	1:2	400	3	60	10

Table S2. The MIC values of PEOs/ZnO/PAL nanocomposites against *E. coli* and *S. aureus.*

**Samples**	**MIC (mg/mL)**	
	E. coli	***S. aureus***
Citral/ZnO/PAL	1.5	5
Thymol/ZnO/PAL	1	2.5
Carvacrol/ZnO/PAL	0.5	1.5
Oregano oil/ZnO/PAL	0.5	2.5
Cinnamaldehyde/ZnO/PAL	1	2.5

Table S3. The MIC values of PAL and ZnO/PAL encompassed by different concentrations of CAR against *E. coli*.

**wt%**	**MIC (mg/mL) against *E. coli***	
	**CAR/PAL**	**CAR/ZnO/PAL**
1	5	1
2.5	5	1
5	2.5	1
10	2.5	0.5
20	1.5	0.5
